# Long-Term Performance and Safety of the Self-Expandable Carotid Stent MER: 5-Year Outcomes from the OCEANUS Study, with Subgroup Analysis Based on Predilatation Before Carotid Artery Stenting

**DOI:** 10.3390/jcm14082814

**Published:** 2025-04-18

**Authors:** Przemysław Nowakowski, Mariola Sznapka, Adam Kobayashi, Jacek Bil, Piotr Paluszek, Eugeniusz Hrycek, Zofia Nowakowska, Michał Nowakowski, Aleksandra Suchanek, Piotr Pieniążek

**Affiliations:** 1 Faculty of Medicine, Academy of Silesia, 40-555 Katowice, Poland; 2 American Heart of Poland, 32-500 Chrzanów, Poland; aleksandra.suchanek@ahop.pl; 3 Faculty of Medicine, Upper Silesian Academy Wojciecha Korfantego, 40-659 Katowice, Poland; m.sznapka@op.pl; 4 Collegium Medicum, Cardinal Stefan Wyszynski University, 01-815 Warsaw, Poland; a.kobayashi@uksw.edu.pl; 5 National Medical Institute of the Ministry of the Interior and Administration, 02-507 Warsaw, Poland; jacek.bil@pimmswia.gov.pl; 6 Department of Vascular Surgery and Endovascular Interventions, St. John Paul II Hospital, 31-202 Krakow, Poland; kardio1@kki.krakow.pl; 7 Department of Cardiology, Faculty of Medical Sciences, Andrzej Frycz Modrzewski Kraków University, 30-705 Kraków, Poland; ehrycek@gmail.com; 8 Faculty of Medicine, University of Silesia, 40-055 Katowice, Poland; nowa.sophie@gmail.com (Z.N.);; 9 Department of Invasive Cardiology, Institute of Cardiology, Jagiellonian University Medical College, 31-202 Krakow, Poland

**Keywords:** carotid artery stenting, MER stent, neuroprotection, predilatation, long-term outcomes, restenosis, major adverse events, Robin

## Abstract

**Background**: Carotid artery stenting (CAS) with neuroprotection is a widely used treatment for carotid artery stenosis. This study aimed to evaluate the long-term outcomes of CAS using the MER stent (Balton, Poland) and various neuroprotection devices, with subgroup analysis based on predilatation. **Methods**: A prospective analysis was conducted on patients treated with CAS at four high-volume centers in Poland between October 2016 and May 2017. Patients were stratified into two groups based on whether predilatation was performed. Procedural and clinical outcomes, including major adverse events (MAEs) defined as all-cause death, stroke, and myocardial infarction (MI), were evaluated at 30 days and 5 years post-procedure. Kaplan–Meier analysis and Cox regression models were used to assess event-free survival and predictors of MAEs. **Results**: The study population consisted of 100 patients (males: 61%) with a mean age of 68 years. Dyslipidemia (84.4% vs. 60.0%, *p* = 0.007) and smoking (67.3% vs. 44.4%, *p* = 0.022) differed significantly between the predilatation and non-predilatation groups. The procedural success rate (<30% residual stenosis) was 97%. At 5 years, the overall restenosis rate was 7%, and target vessel revascularization was required in 3% of patients. The cumulative mortality rate was 15%, and two strokes (2%) were recorded. Multivariable regression identified prior CABG as an independent predictor of MAEs (HR 3.5, 95% CI 1.14–10.83, *p* = 0.03). **Conclusions**: CAS with the MER stent demonstrated high procedural success and favorable long-term outcomes. Predilatation did not impact outcomes. Neuroprotection was effective in all cases, with no device-related complications reported.

## 1. Introduction

Stroke remains a major public health concern in the United States, ranking as the leading cause of disability and the fifth leading cause of mortality, with approximately 140,000 deaths annually. Ischemic strokes, which constitute nearly 87% of all stroke cases, contribute significantly to this burden. The economic impact is substantial, with stroke-related costs estimated at USD 56.5 billion between 2018 and 2019, projected to rise to USD 94.3 billion by 2035. Carotid artery stenosis accounts for 10–20% of all ischemic strokes. A 2020 meta-analysis reported a global prevalence of extracranial carotid stenosis >50% in 1.8% of men and 1.2% of women, with prevalence increasing with age [1,2]. Also, in Europe, stroke incidence and prevalence have also risen, with the number of new cases and stroke-related deaths increasing by 2–6% across the region between 2010 and 2019 [3].

Carotid endarterectomy (CEA) has long been the gold standard for treating severe carotid artery stenosis. However, over the past 30 years, the development of minimally invasive transfemoral carotid artery stenting (CAS) has offered a less invasive alternative to traditional surgery. Despite its advantages, concerns have been raised about the increased risk of periprocedural stroke associated with CAS [4,5]. Also, long-term data are essential to fully understand the durability and safety of CAS, particularly with modern self-expanding stents designed to optimize vessel patency and reduce procedural complications [6,7].

The MER carotid self-expanding stent, manufactured from nitinol, has demonstrated promising performance in maintaining lumen integrity and minimizing restenosis rates. While previous studies have focused on short-term outcomes, the long-term performance of CAS using the MER stent remains less well documented [8,9]. Here, we present the 5-year follow-up data from a multicenter study evaluating the safety and efficacy of the MER stent, with a specific focus on the incidence of major adverse events (all-cause death, stroke, and myocardial infarction [MI]) and subgroup analyses based on the use of predilatation before stent deployment. This analysis provides valuable insights into the long-term outcomes of CAS with the MER stent and its role in managing carotid artery stenosis.

## 2. Materials and Methods

### 2.1. Study Design and Patient Qualification

Between October 2016 and May 2017, 100 patients underwent CAS with various protection devices using a self-expanding nitinol stent (MER). The study was conducted across four centers in Poland that specialize in CAS. It was approved by the Ethics Committee at the Beskid Medical Chamber in Bielsko-Biala, Poland (opinion number: 2015/03/26/2, dated 26 March 2015). It was registered at ClinicalTrials.gov (NCT03133429). All patients provided written informed consent and were evaluated with a Doppler ultrasound of the carotid arteries, with angio-CT of the aortic arch and brain-supplying arteries performed at the operator’s discretion. Eligible patients included those with symptomatic internal carotid artery (ICA) stenosis of ≥50% or asymptomatic stenosis of ≥75%, as determined by a neurologist. Neurological assessments were performed by the same neurologist before the procedure, immediately after CAS, at discharge, and 30 days post procedure. Periprocedural dual antiplatelet therapy (aspirin 75 mg/day and clopidogrel 75 mg/day) was administered for at least three days before CAS. Post-procedural therapy consisted of clopidogrel and aspirin for three months and lifelong aspirin. Detailed inclusion and exclusion criteria are provided in the Supplementary Table S1.

### 2.2. Device and Procedural Details

The MER carotid self-expanding stent, manufactured by Balton (Warsaw, Poland), is composed of an open-cell nitinol alloy and shaped using laser technology. The stent is mounted on the distal end of a rapid-exchange 0.014-inch delivery system and self-expands into a cylindrical shape upon release, restoring the optimal lumen geometry of the vessel. The stent is positioned between two markers at the distal tip of the delivery system for precise deployment. The stent is available as a straight or tapered stent and is compatible with a 5F introducer. It is available in diameters from 4 to 10 mm and lengths from 20 to 60 mm.

On the day of the procedure, hypotensive, diuretic, and anti-arrhythmic medications were withheld, and all patients received 500 mL of isotonic saline before and during the intervention. All procedures were performed from the femoral access. Cerebral protection devices were used in all cases. Self-expanding, open-cell nitinol MER stents were implanted in all patients. Predilatation and postdilatation were at the operator’s discretion.

Patients received unfractionated heparin during the procedure to maintain an activated clotting time of 250–300 s. Additionally, patients without implanted pacemakers received 0.5–1.0 mg of atropine before stent deployment. Pre- and post-procedural intracranial angiography was performed for all patients to identify and exclude periprocedural embolic complications. Following femoral artery angiography, arterial puncture sites were typically closed using manual compression or vascular closure devices.

### 2.3. Follow-Up

Ultrasound examinations were conducted before discharge and at 30 days, 6 months, and 12 months to evaluate the implanted stent. Neurological consultations were performed at baseline and at 30-day and 1-year follow-ups to assess any neurological incidents that occurred during the observation period. Data on the occurrence of MI and mortality were collected. An additional 5-year telephone follow-up was performed.

### 2.4. Study Endpoints

The primary endpoint was the cumulative incidence of major adverse events (MAEs) at 30 days and 1 year, defined as all-cause death, stroke, and MI. This report extends the analysis to 5-year outcomes, including a subgroup analysis based on the use of predilatation before CAS.

### 2.5. Statistics

Categorical variables are presented as numbers and percentages, while continuous variables are expressed as mean ± standard deviation or median and interquartile range. Normality was assessed using the Shapiro–Wilk test, and equality of variances was evaluated using Levene’s test. Differences between groups were assessed using Student’s *t*-test or Welch’s *t*-test, depending on variance equality for normally distributed variables. The Mann–Whitney U-test was applied for non-normally distributed continuous variables. Ordinal variables were compared using the Cochran–Armitage test for trend, while nominal variables were compared using Pearson’s chi-squared test or Fisher’s exact test when expected counts in more than 20% of cells were less than 5.

Event-free survival was analyzed using Kaplan–Meier curves, with differences between groups tested using the log-rank statistic. Determinants of major adverse events (MAEs) were assessed through univariable and multivariable Cox regression models. Multivariable models were fitted using backward stepwise regression, with a *p*-value threshold of 0.1 for the stopping rule. Variables considered for inclusion in the multivariable logistic regression model included baseline demographic characteristics (e.g., age, sex, smoking status, hypertension, dyslipidemia, prior transient ischemic attack [TIA], MI, percutaneous coronary intervention [PCI], or coronary artery bypass grafting [CABG]), lesion characteristics (% diameter stenosis [%DS] before procedure, lesion length, and lesion localization [common/internal]), periprocedural data (neuroprotection [proximal/distal] and predilatation), and baseline lab data.

Statistical analyses were performed using JMP^®^, Version 17.2.0 (SAS Institute Inc., Cary, NC, USA) and R, Version 3.4.1 (R Core Team, Vienna, Austria, 2017; https://www.r-project.org/, accessed on 16 November 2024).

## 3. Results

### 3.1. Population

The study was performed in four high-volume centers, and 100 patients were included. The mean age of the population was 68.43 years, with no significant difference between the “without predilatation” group (67.62 ± 8.07 years, *n* = 55) and the “with predilatation” group (69.42 ± 8.31 years, *n* = 45; *p* = 0.275). Males constituted 61% of the overall cohort (*n* = 61), with a slightly higher prevalence in the “without predilatation” group (65.45%, *n* = 36) compared to the “with predilatation” group (55.56%, *n* = 25; *p* = 0.312).

Significant differences were observed in the prevalence of dyslipidemia and smoking. Dyslipidemia was more common in the “with predilatation” group (84.44%, *n* = 38) compared to the “without predilatation” group (60.00%, *n* = 33; *p* = 0.007). Conversely, smoking was significantly more frequent in the “without predilatation” group (67.27%, *n* = 37) compared to the “with predilatation” group (44.44%, *n* = 20; *p* = 0.022). Other characteristics, such as the proportion of symptomatic patients (44% overall, *n* = 44; 52.73%, *n* = 29 in the “without predilatation” group vs. 33.33%, *n* = 15 in the “with predilatation” group; *p* = 0.052) and the prevalence of arterial hypertension (91% overall, *n* = 91; 92.73%, *n* = 51 vs. 88.89%, *n* = 40; *p* = 0.728), were comparable between groups. There were no significant differences in prior cardiovascular events (e.g., prior TIA, stroke, MI, PCI, or CABG) or renal function as assessed by eGFR (*p* = 0.855) (Table 1).

### 3.2. Procedural Data

The procedural details of CAS with the MER stent were consistent with standard clinical practice, focusing on optimizing outcomes through careful lesion preparation and neuroprotection. The procedures were performed predominantly via the right access site in 95% of patients (*n* = 95). Lesions were located in the internal carotid artery in 95% of cases (*n* = 95), with a smaller proportion involving the common carotid artery (5%, *n* = 5). The mean lesion length was 17.22 ± 8.15 mm, and the mean reference vessel diameter was 5.65 ± 0.93 mm. Postdilatation was performed in all cases (100%, *n* = 100), ensuring optimal stent expansion.

The procedural success rate, defined as achieving <30% residual stenosis without major complications, was high at 97% (*n* = 97). In three patients, residual %DS was larger than 30%: 32% (in this patient, a second stent was implanted), 39%, and 55%.

Single-stent implantation was sufficient in 97% of patients (*n* = 97), while two stents were required in 3% (*n* = 3). The reasons for the second stent deployment were as follows: distal dissection (*n* = 1), first stent migration into the common carotid artery (*n* = 1), and not completely covering the long lesion (*n* = 1).

Neuroprotection devices were used in all procedures, reflecting a strong focus on minimizing embolic complications during CAS. The distribution of neuroprotection devices included Filter Wire EZ (31%, *n* = 31; Boston Scientific, Marlborough, MA, USA), Spider FX (28%, *n* = 28; Medtronic, Minneapolis, MN, USA), Mo.Ma (19%, *n* = 19; Medtronic, Minneapolis, MN, USA), Robin (10%, *n* = 10; Balton, Warsaw, Poland), Wirion (7%, *n* = 7; Gardia Medical, Caesarea, Israel), and Emboshield (5%, *n* = 5; Abbott Vascular, Santa Clara, CA, USA). Debris in the protection devices was estimated in one center. Embolic material was found in 15/47 (32%) patients. No complications related to the neuroprotection systems were reported.

These results underscore the importance of procedural strategies, including the routine use of neuroprotection devices, in ensuring the safety and efficacy of CAS with the MER stent. The high procedural success rate highlights the effectiveness of these measures in achieving favorable outcomes (Table 2).

### 3.3. Follow-Up

**Figure 1:**
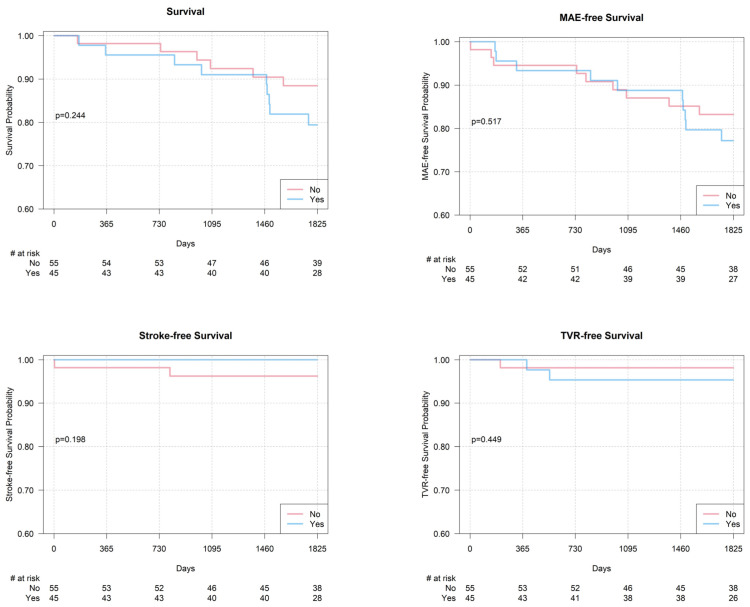
Kaplan–Meier curves showing 5-year event-free survival depending on predilatation (NO—no predilatation; YES—predilatation performed) before carotid artery stenting: all-cause death; major adverse events; stroke; target vessel revascularization; #: number.

A major stroke occurred on the fourth day post-procedure in a high-risk symptomatic patient with a history of contralateral internal carotid artery occlusion (symptomatic subgroup—2.3%; the whole population—1%). The patient experienced aphasia lasting over 24 h, which resolved with neurological treatment, and other symptoms subsided five days after the stroke. Within 30-day follow-up, no additional adverse events were reported. During 5-year follow-up, 15 deaths (15%) were registered, including sudden deaths (*n* = 5), acute cardiopulmonary insufficiency (*n* = 3), lung cancer (*n* = 2), COVID-19 infection (*n* = 2), complications after limb ischemia treatment (*n* = 2), and suicide (*n* = 1). The restenosis rate in a 5-year follow-up was 7%, and the target vessel revascularization rate was 3% (one drug-eluting balloon and two bare metal stents) (Table 3, Figure 1).

### 3.4. Regression Analysis

The univariable Cox regression analysis results are presented in Supplementary Table S2. Based on the multivariable regression analysis, the only independent predictor of MAEs was prior CABG (HR 3.5, 95% CI 1.14–10.83, *p* = 0.03) (Table 4).

## 4. Discussion

This study provides valuable insights into the long-term outcomes of carotid artery stenting (CAS) with the MER stent, focusing on the impact of predilatation and the role of neuroprotection devices. The high procedural success rate (97%) and low rates of restenosis (7%) as well as target vessel revascularization (3%) observed at 5 years, are consistent with the outcomes reported in other studies evaluating modern self-expanding nitinol stents in CAS. These findings underscore the efficacy of the MER™ stent in restoring vessel patency and maintaining long-term durability while ensuring procedural safety through the use of neuroprotection devices.

Comparing our findings to the CREST, which remains a landmark study in the field, highlights several important parallels [10]. The CREST reported 4-year stroke rates of 6.4% for CAS and 4.7% for carotid endarterectomy (CEA), with comparable restenosis rates between the two interventions over extended follow-up periods. In our study, the 5-year stroke rate was only 2%, which supports the safety of the MER™ stent, particularly when paired with neuroprotection devices. This low stroke rate aligns with other reports emphasizing the critical role of neuroprotection devices in minimizing embolic risks during CAS [11].

Our findings align with the current European Society for Vascular Surgery (ESVS) and European Society of Cardiology (ESC) guidelines, which advocate for individualized approaches to carotid revascularization [12,13]. The 2023 ESVS guidelines on the management of carotid artery disease recommend carotid artery stenting (CAS) as a Class IIa indication for symptomatic patients at high surgical risk for carotid endarterectomy (CEA), particularly when performed by experienced operators in high-volume centers equipped with modern neuroprotection devices. The guidelines emphasize using neuroprotection, such as embolic protection devices, as a standard practice to reduce the risk of periprocedural stroke (class I recommendation). Additionally, predilatation and postdilatation strategies should be carefully considered and tailored to the individual patient’s anatomy and clinical context, with particular caution in cases of heavily calcified plaques or high embolic risk [14,15].

The subgroup analysis comparing patients with and without predilatation revealed that predilatation was associated with longer lesion lengths and higher rates of dyslipidemia, reflecting its use in more complex lesions. Despite these differences, there was no significant impact on restenosis or MAEs, suggesting that predilatation can be selectively applied based on lesion and patient characteristics. This finding aligns with the guidelines, which advocate tailoring procedural techniques, including predilatation, to individual anatomical and clinical factors [12,13].

Neuroprotection played a crucial role in ensuring procedural safety, with a range of devices employed, including Filter Wire EZ, Spider FX, Mo.Ma, Robin, Wirion, and Emboshield. Each device was selected based on anatomical and procedural considerations, reflecting real-world clinical practice. Importantly, no device-related complications were observed, and embolic material was identified in 32% of patients, emphasizing the effectiveness of these devices in capturing debris and preventing distal embolization. The absence of device-related complications further supports the safety of neuroprotection systems, as highlighted in other studies, including those by Cremonesi et al., which demonstrated significant reductions in embolic stroke with proximal protection devices [16]. However, distal neuroprotection systems are currently the mainstay [17,18,19].

Regarding periprocedural stroke rates, studies have reported varying outcomes. For instance, the Carotid Revascularization Endarterectomy vs. Stenting Trial (CREST) reported a periprocedural stroke rate of 4.1% for patients who underwent CAS. Contemporary cohort studies have reported stroke rates ranging from 1.1% to 3.2% [20]. Moreover, the results obtained in the OCEANUS study met the acceptable rates of peri-procedural complications (including death, stroke, and MI) for CAS in symptomatic patients (6%) as well as asymptomatic patients (3%) published by the Society for Vascular Surgery in guidelines in 2024 [21].

The restenosis rate of 7% at 5 years, observed in this study, is comparable to other studies evaluating nitinol stents, such as the SPACE trial, the SAPPHIRE trial, or the ACT I study, which reported long-term restenosis rates between 5% and 10% [22,23,24]. In our cohort, restenosis cases were successfully managed with minimal reintervention, suggesting the effectiveness of the MER stent design in minimizing restenosis and maintaining vessel patency. The routine use of postdilatation in all cases likely contributed to the durability of stent outcomes by ensuring optimal stent expansion and reducing the risk of plaque prolapse.

In a recent study, ACST-2, the results were similar. Between 15 January 2008 and 31 December 2020, a total of 3625 patients from 130 centers were randomly assigned to carotid artery stenting (CAS; *n* = 1811) or carotid endarterectomy (CEA; *n* = 1814), with good adherence to the protocol, optimal medical therapy, and an average follow-up of 5 years. Procedural outcomes showed that 1% of patients experienced disabling stroke or death (15 in the CAS group and 18 in the CEA group), while 2% experienced non-disabling procedural stroke (48 in the CAS group and 29 in the CEA group). Kaplan–Meier estimates for 5-year non-procedural stroke revealed similar rates of fatal or disabling stroke in both groups (2.5%), while the rates for any stroke were 5.3% with CAS and 4.5% with CEA (rate ratio [RR] 1.16, 95% CI 0.86–1.57; *p* = 0.33). A meta-analysis of rate ratios for any non-procedural stroke across all CAS versus CEA trials found no significant differences between symptomatic and asymptomatic patients, with an overall RR of 1.11 (95% CI 0.91–1.32; *p* = 0.21) [25]. These results are more favorable compared to the results of a recent meta-analysis [26].

Moreover, Hajiyev et al. published results for over 10 years [27]. Between 2009 and 2020, 1158 patients (636 asymptomatic and 522 symptomatic) underwent CAS. Long-term outcomes were assessed via telephone interviews with 560 patients or contacts (316 asymptomatic and 244 symptomatic), with a mean follow-up of 5 years. Overall survival rates were 91.6% at 1 year, 77.1% at 5 years, and 55.7% at 10 years, while stroke-free survival rates were 97.9%, 92.7%, and 86.6%, respectively. Ischemic stroke occurred in 6.9% of patients during follow-up, with no significant differences in survival outcomes between symptomatic and asymptomatic groups.

The multivariable regression analysis identified prior coronary artery bypass grafting (CABG) as an independent predictor of MAEs, with a hazard ratio of 3.5. This finding aligns with prior studies that have identified CABG as a risk factor for worse outcomes in CAS, likely due to the complex cardiovascular profile of these patients. This underscores the importance of individualized risk assessment and careful procedural planning for high-risk subgroups, particularly those with significant comorbidities.

### Study Limitations

This study has several limitations that should be acknowledged. First, the sample size was relatively small (*n* = 100), which may limit the generalizability of the findings to broader populations. Second, while the study was conducted across four high-volume centers, variations in operator experience and procedural techniques may have influenced outcomes. Third, the absence of randomization and the observational nature of the study may introduce selection bias, particularly in the decision to perform predilatation. Fourth, the long-term follow-up data were derived from a single cohort, and the potential for unmeasured confounding factors cannot be excluded. Also, while neuroprotection devices were used in all cases, the selection of devices was left to the operator’s discretion, which may limit the ability to draw definitive conclusions about the relative efficacy of different devices. Finally, this was a multicenter study conducted across four high-volume centers, and although all operators were experienced in carotid interventions, some variability in procedural techniques and decision-making (e.g., use of predilatation or type of neuroprotection device) may have influenced outcomes. However, the high procedural success rate and consistency of follow-up across sites suggest that these differences did not significantly impact the overall findings.

## 5. Conclusions

This study demonstrates the long-term safety and efficacy of the MER self-expanding nitinol stent in carotid artery stenting, with high procedural success, low restenosis, and minimal reintervention over 5 years. The use of neuroprotection devices contributed to low stroke rates without device-related complications.

Predilatation was used in more complex cases but did not impact long-term outcomes, supporting its selective use. Prior CABG was identified as a predictor of adverse events, emphasizing the need for individualized strategies. These findings help fill a gap in long-term data for modern nitinol stents and support their use in experienced centers. Further trials are needed to validate these results.

## Figures and Tables

**Table 1:** Baseline demographics.

Parameter	Whole Population N = 100 (%)	Without Predilatation N = 55 (%)	With Predilatation N = 45 (%)	*p*
Age [years]	68.43 ± 8.18	67.62 ± 8.07	69.42 ± 8.31	0.275
Sex: males [%]	61 (61.00)	36 (65.45)	25 (55.56)	0.312
Symptomatic patients	44 (44.00)	29 (52.73)	15 (33.33)	0.052
Arterial hypertension	91 (91.00)	51 (92.73)	40 (88.89)	0.728
Dyslipidemia	71 (71.00)	33 (60.00)	38 (84.44)	0.007
Diabetes	39 (39.00)	19 (34.55)	20 (44.44)	0.312
Smoking	57 (57.00)	37 (67.27)	20 (44.44)	0.022
Prior transient ischemic attack	19 (19.00)	12 (21.82)	7 (15.56)	0.427
Prior stroke	43 (43.00)	24 (43.64)	19 (42.22)	0.887
Prior myocardial infarction	25 (25.00)	15 (27.27)	10 (22.22)	0.562
Prior PCI	32 (32.00)	20 (36.36)	12 (26.67)	0.301
Prior CABG	13 (13.00)	9 (16.36)	4 (8.89)	0.269
eGFR [mL/min/1.73 m^2^]	76.00 (53.15; 89.00)	72.00 (54.00; 86.00)	73.00 (54.00; 86.75)	0.855

Results are presented as number (%); mean ± standard deviation, or median (min–max). PCI—percutaneous coronary intervention; CABG—coronary artery bypass grafting; eGFR—estimated glomerular filtration rate.

**Table 2:** Procedural data.

Parameter	Whole Population N = 100 (%)	Without Predilatation N = 55 (%)	With Predilatation N = 45 (%)	*p*
%DS before CAS *	76.29 ± 9.63	75.19 ± 10.49	77.62 ± 8.38	0.211
%DS just after CAS *	15.31 ± 9.89	12.52 ± 8.70	10.24 ± 6.86	0.007
%DS at discharge *	12.72 ± 9.68	11.64 ± 10.20	14.03 ± 8.95	0.087
%DS at discharge **	36.02 ± 23.21	35.13 ± 24.98	38.50 ± 18.25	0.934
Access site: right	95 (95.00)	50 (90.91)	45 (100.00)	0.062
Lesion location				0.655
Internal	95 (95.00)	53 (96.36)	42 (93.33)	
Common	5 (5.00)	2 (3.64)	3 (6.67)	
Reference vessel diamater [mm]	5.65 ± 0.93	5.92 ± 0.87	5.32 ± 0.89	<0.001
Lesion length [mm]	17.22 ± 8.15	15.71 ± 7.86	19.06 ± 8.20	0.036
No of predilatation				-
1	-	42 (93.33)	-	
2	-	2 (4.44)	-	
3	-	1 (2.22)	-	
Number of implanted stents				0.587
1	97 (97.00)	54 (98.18)	43 (95.56)	
2	3 (3.00)	1 (1.82)	2 (4.44)	
Stent diameter [mm]				0.063
7	1 (1.00)	1 (1.82)	0 (0.00)	
8	8 (8.00)	1 (1.82)	7 (15.56)	
9	3 (3.00)	1 (1.82)	2 (4.44)	
6 × 8	56 (56.00)	35 (63.64)	21 (46.67)	
7 × 9	18 (18.00)	7 (12.73)	11 (24.44)	
7 × 10	10 (10.00)	7 (12.73)	3 (6.67)	
8 × 10	4 (4.00)	3 (5.45)	1 (2.22)	
Stent length [mm]				0.407
20	3 (3.00)	1 (1.82)	2 (4.44)	
30	52 (52.00)	28 (50.91)	24 (53.33)	
40	44 (44.00)	25 (45.45)	19 (42.22)	
50	1 (1.00)	1 (1.82)	0 (0.00)	
Neuroprotection				0.007
Emboshield	5	3	2	
Filter Wire EZ	31	28	3	
Mo.Ma	19	6	13	
Robin	10	2	8	
Spider FX	28	12	16	
Wirion	7	4	3	
Postdilatation	100 (100)	55 (100.00)	45 (100.00)	1.0
Procedural success	97 (97.00)	55 (100.00)	42 (93.33)	0.088

* %DS calculated based on the NASCET formula; ** %DS calculated based on the ECST formula; %DS—diameter stenosis; CAS—carotid artery stenting.

**Table 3:** 5-year clinical outcomes.

Parameter	Whole Population N = 100 (%)	Without Predilatation N = 55 (%)	With Predilatation N = 45 (%)	*p*
Death	15 (15.00)	6 (10.91)	9 (20.00)	0.205
Stroke	2 (2.00)	2 (3.64)	0 (0.00)	0.500
Hemorrhagic	1 (1)	1 (1.82)	0 (0.00)	1.000
Ischemic	1 (1)	1 (1.82)	0 (0.00)	1.000
Myocardial infarction	4 (4.00)	1 (1.82)	3 (6.67)	0.324
Restenosis (%DS ≥ 50%)	7 (7.00)	3 (5.45)	4 (8.89)	0.697
Target vessel revascularization	3 (3.00)	1 (1.82)	2 (4.44)	0.587

**Table 4:** Multivariable regression analysis.

Parameter	Hazard Ratio	Lower 95%	Upper 95%	*p*
Predilatation	2.654	0.915	7.692	0.072
Symptomatic patient	2.542	0.935	6.911	0.067
Prior CABG	3.504	1.144	10.731	0.028
Age	1.057	0.994	1.128	0.084
Platelet value	0.992	0.982	0.999	0.052

## Data Availability

On request from the corresponding author.

## Supplementary Materials

The following supporting information can be downloaded at: https://www.mdpi.com/article/10.3390/jcm14082814/s1, Supplementary Table S1: Inclusion and exclusion criteria; Supplementary Table S2: Univariable regression analysis for MAE.

## Abbreviations

The following abbreviations are used in this manuscript:

%DS	Percent diameter stenosis
ACST-2	Asymptomatic Carotid Surgery Trial 2
CABG	Coronary artery bypass grafting
CAS	Carotid artery stenting
CEA	Carotid endarterectomy
CI	Confidence interval
eGFR	Estimated glomerular filtration rate
HR	Hazard ratio
MAE	Major adverse event
MI	Myocardial infarction
NASCET	North American Symptomatic Carotid Endarterectomy Trial
PCI	Percutaneous coronary intervention
TIA	Transient ischemic attack
UI	Uncertainty interval
