# Supplementary material for: Long-Term Performance and Safety of the Self-Expandable Carotid Stent MER: 5-Year Outcomes from the OCEANUS Study, with Subgroup Analysis Based on Predilatation Before Carotid Artery Stenting
Source: J Clin Med. 2025 Apr 18;14(8):2814. doi: 10.3390/jcm14082814 (PMC12027988; doi:10.3390/jcm14082814)
Supplement: Supplementary file 1 [file jcm-14-02814-s001.zip › jcm-3488668-supplementary.pdf]

Supplementary Table S1. Inclusion and exclusion criteria.

INCLUSION CRITERIA

1. De-novo lesion in the common or internal carotid artery
2. %DS ≥50% (symptomatic patients) and ≤99% based on QCA analysis
3. %DS ≥75% (asymptomatic patients) and ≤99% based on QCA analysis
4. The lesion can be covered with a stent 20–60 mm in length and 4–10 mm in diameter (including tapered stents)
5. Patient eligible for CAS (Carotid Artery Stenting)
6. Age ≥18 years
7. Life expectancy >12 months from the procedure
8. Written informed consent obtained from the patient prior to the procedure

EXCLUSION CRITERIA

1. Lack of neurological qualification for the procedure
2. Myocardial infarction within 72 hours before the procedure
3. Paroxysmal, persistent, or permanent atrial fibrillation or flutter
4. Active gastrointestinal bleeding
5. Pregnancy
6. Contraindications to dual antiplatelet therapy
7. Planned surgical procedure within 1 month of the investigational stent implantation
8. Platelet count <100,000/mm^3 or >600,000/mm^3
9. Known hypersensitivity to nickel, titanium, or contrast agents
10. Occlusion of the target vessel
11. Previously implanted stent in the target vessel
12. Contraindications to statin therapy
13. Heavy calcifications at the stenosis site
14. Hyperthyroidism
15. Lesions resulting from prior radiotherapy
16. Absence of palpable femoral pulses
17. Renal failure (serum creatinine >2.0 mg/dL or eGFR <30 mL/min/1.73 m^2 or patients on dialysis)

Supplementary Table S2. Cox Regression – univariable analysis.

Variable	HR per	HR (95% CI)	p-value	# of events	# of censorings
Predilatation	No/Yes	0.743 (0.302; 1.830)	0.5187	19	81
Patient Status	Asymptomatic/Symptomatic	0.653 (0.265; 1.607)	0.3531	19	81
Sex	Female/Male	0.727 (0.276; 1.913)	0.5185	19	81
Hypercholesterolemia (screening)	No/Yes	0.831 (0.299; 2.307)	0.7219	19	81
Family History of Stroke (screening)	No/Yes	2.441 (0.326; 18.289)	0.3850	19	81
Smoking (screening; categorized)	No/Yes, current or past	0.933 (0.375; 2.320)	0.8816	19	81
Diabetes Mellitus (screening)	No/Yes	0.872 (0.351; 2.169)	0.7690	19	81
Hypertension (screening)	No/Yes	0.496 (0.066; 3.718)	0.4953	19	81
Previous TIA (screening)	No/Yes	1.161 (0.338; 3.984)	0.8130	19	81
Previous Stroke (screening)	No/Yes	0.810 (0.329; 1.993)	0.6463	19	81
Time Since Last Stroke (screening)	≥12 months / <6 months	0.478 (0.060; 3.820)	0.4861	9	33
Stroke Side Consistency (screening)	No/Yes	0.604 (0.126; 2.910)	0.5301	9	34
Stroke Symptoms: Hemiparesis or Paralysis (screening)	No/Yes	0.415 (0.052; 3.320)	0.4072	9	33

Previous Myocardial Infarction (screening)	No/Yes	0.451 (0.181; 1.122)	0.0867	19	81
Previous PCI (screening)	No/Yes	0.518 (0.211; 1.275)	0.1524	19	81
Previous CABG (screening)	No/Yes	0.392 (0.141; 1.089)	0.0725	19	81
Periprocedural Complications (CAS)	No/Yes	66840673.844 (0.000; .)	0.9992	19	81
Access Site	Left/Right	8.881 (2.517; 31.343)	0.0007*	19	81
Target Lesion Location (Carotid Artery)	Internal/Common	0.886 (0.118; 6.642)	0.9066	19	81
Target Lesion Location (Side)	Left/Right	0.824 (0.324; 2.093)	0.6839	19	81
Neuroprotection System	Distal/Proximal	0.855 (0.284; 2.576)	0.7803	19	81
Need for Second Stent (CAS)	No/Yes*	0.250 (0.058; 1.088)	0.0646	19	81
Age at Admission	1 year	1.053 (0.995; 1.116)	0.0734	19	81
Systolic Blood Pressure at Admission (screening)	1 mmHg	1.008 (0.987; 1.028)	0.4462	19	81
Diastolic Blood Pressure at Admission (screening)	1 mmHg	1.004 (0.962; 1.044)	0.8623	19	81
PLT Value (screening)	1 x 1000/µL	0.992 (0.983; 1.000)	0.0459*	19	81
RBC Value (screening)	1 x 1,000,000/mm^3	0.468 (0.173; 1.278)	0.1382	19	81
Stenosis Percentage (max) by NASCET (screening)	1%	0.999 (0.954; 1.050)	0.9822	19	81
Stenosis Percentage Before Procedure (max)	1%	0.986 (0.938; 1.038)	0.5830	19	81

Stenosis Percentage Immediately After Procedure (max)	1%	1.052 (1.009; 1.087)	0.0192*	19	81
Stenosis Percentage (max) by NASCET (at discharge)	1%	1.019 (0.974; 1.060)	0.3951	19	81
Lesion Length, mm	1 mm	1.016 (0.962; 1.067)	0.5427	19	81
eGFR (screening), mL/min/1.73 m^2	1 mL/min/1.73 m^2	0.964 (0.936; 0.990)	0.0071*	16	64
